# Core binding factor acute myelogenous leukemia-2021 treatment algorithm

**DOI:** 10.1038/s41408-021-00503-6

**Published:** 2021-06-16

**Authors:** Gautam Borthakur, Hagop Kantarjian

**Affiliations:** Department of Leukemia, MD Anderson Cancer Center, Houston, TX USA

**Keywords:** Chemotherapy, Chemotherapy

## Abstract

Core binding factor acute myelogenous leukemia (CBF-AML), characterized by the presence of either t(8;21) (q22;q22) or inv(16) (p13q22)/t(16;16), is considered good-risk AML in the context of cytarabine based intensive chemotherapy. Still, outcome can be improved significantly through the effective implementation of available therapeutic measures and appropriate disease monitoring. The incorporation of gemtuzumab ozogamicin into frontline therapy should be standard. Cytarabine based induction/consolidation regimen may be combined with anthracycline (3 + 7 standard) or antimetabolite, fludarabine. Serial quantitative polymerase chain reaction (QPCR) monitoring of unique fusion transcripts allows monitoring for measurable residual disease clearance; this allows for better prognostication and well as treatment modifications.

## Background

Core-binding factor (CBF) acute myeloid leukemia (AML), comprising up to 12–15% of all AML cases [1–5], is characterized by the presence of either t(8;21)(q22;q22) or inv(16)(p13q22)/t(16;16), which leads to the formation of *RUNX1/RUNX1T1 (AML1/ETO)* [6] and *CBFB/MYH11* [7] fusion genes respectively. These cytogenetic aberrations are associated with favorable response and increased sensitivity to cytarabine. However, long-term follow-up reports from larger groups show a median overall survival (OS) of ~5 years or less [1, 8–10], indicating the need to improve therapy further. The population-based SEER data from 2000 to 2010 reports a 3-year OS rate of 57.3% for patients with inv(16), but only 35.5% in t(8;21).This suggests the possibility of substantial improvement of outcome in both AML entities.

## Pathogenesis

Core binding factors are hematopoietic transcription factors characterized by heterodimers of two units: a DNA binding unit, and a CBFA and non-DNA binding unit, CBFB (reviewed in Speck and Gilliland [11]). CBFA comprises of three subunits; RUNX1, RUNX2, and RUNX3, while CBFB is invariate and is the non-DNA binding unit of the heterodimer. Core binding factors are necessary in normal hematopoiesis. Translocation events alter the normal DNA binding of the heterodimer and create alternate binding, leading to disruption of the normal transcription program and resulting in maturation arrest [12]. For example, the fusion protein AML/ETO negatively impacts the myeloid differentiation promoting transcriptional effect of PU1 by binding and displacing the coactivator c-Jun from PU1 [13].

The unique translocation event is likely not enough for leukemogenesis and additional mutational events involving epigenetic/chromatin modulators (e.g. *TET2, ASXL1, ASXL2*), kinases (e.g. *FLT3, RAS*) and others are needed for leukemic development and progression [14, 15]. The overall mutational burden is however lower in CBF AML compared to intermediate or high risk AML [14, 16].

## t(8;21) and inv16 AML- are they two different diseases?

Though both t(8;21) and inv16 AML involve CBF translocations and are considered good-risk AML, outcomes of t(8;21) and inv16 are quite different. While remission rates are similarly high, relapses are more frequent in the t(8;21)AML and long-term outcome is worse [17]. The co-operating mutational profiles are also different: t(8;21) AML is associated more often with epigenetic/chromatin modulator/cohesion mutations while inv16 AML is associated more often with kinase mutations [14, 16, 18], particularly MAPK pathway mutations. Epigenetic mutations are relatively infrequent in inv16 AML.

Interestingly, multiple mutations in the same signaling pathway are frequent in CBF AML, which indicates clonal interference or parallel evolution. The presence of clonal interference does not impact remission or measurable residual disease (MRD) clearance, but may be associated with inferior event-free survival (EFS) [19].

## Optimizing remission induction therapy in CBF AML

Remission rates are usually high in CBF AML. Remission induction with cytarabine/anthracycline-based chemotherapy combinations and consolidation with high-dose cytarabine (HDAC)-based regimens is considered as the standard of care for AML. However, in addition to complete morphological remission (CR), the depth of remission is important as several studies have shown that early reduction in the CBF related fusion transcript translates into better relapse-free survival (RFS) [20–22].

Fludarabine, cytarabine and GCSF (FLAG)-based regimens can be effective alternative to the traditional “3 + 7” based approach [23–25]. CBF AML is considered sensitive to cytarabine and the sequential administration of fludarabine followed by cytarabine allows increased ara-CTP accumulation in AML blasts [26]. This rationale led to the investigation of adding fludarabine to cytarabine based regimens with or without anthracycline in CBF AML.

We reported an analysis of non-randomized data from 114 patients with CBF AML treated at MD Anderson with the following induction regimens: (1) fludarabine and cytarabine (FA) (*n* = 45); (2) FA with G-CSF (FLAG) (*n* = 22); and (3) idarubicin and cytarabine with or without GCSF (IA or IAG) (*N* = 47). A multivariate analysis showed that the FLAG regimen was associated with longer event-free survival (EFS) than IA/IAG with a relative risk (RR) of 0.47 (FLAG vs. IA/IAG; *p* = 0.07). The EFS was similar with FA and IA/IAG [RR = 0.84 FA vs. IA/IAG, *p* = 0.58)]. A post hoc analysis of the Medical research Council (MRC) AML15 randomized trial showed that patients with CBF AML who completed two courses of FLAG and idarubicin (FLAG-IDA) followed by 2 courses of HDAC had the best long term survival [25, 27].

## Addition of gemtuzumab ozogamicin to remission induction therapy

A meta-analysis of five randomized trials showed that the addition of Gemtuzumab Ozogamicin (GO) to remission induction therapy improved survival in CBF AML, with an absolute survival benefit of 20.7% (OR 0.47, 0.31–0.73; *p* = 0.0006) [28], even though remission rates were not higher with GO containing regimens. The largest benefits were observed in the studies that used lower doses of GO (3 mg/m^2^: MRC trial) or fractionated doses of GO (3 mg/m^2^ on Days 1, 3, and 5: ALFA trial) compared with GO 6 mg/m^2^, We reported 3-year OS and RFS rates of 78% and 85% respectively in an ongoing study of FLAG-GO (3 mg/m^2^ of GO single dose in induction) [23]. Thus, the incorporation of GO into the remission induction should be considered standard for CBF AML.

## Optimizing the post-remission therapy in CBF AML

A retrospective analysis of four successive CALGB trials included 50 patients with t(8;21) [29]. As consolidation, either three cycles or more of HDAC or one cycle of HDAC was administered, followed by additional non-cytarabine based post-remission therapy. Consolidation therapy consisting of 3 or 4 cycles of HDAC (*n* = 21) was associated with a lower relapse rate and a better OS compared with one course of HDAC (*n* = 29). A similar report from the CALGB group that included 48 patients with inv(16)/t(16;16) AML under 60 years showed also a significantly lower 5-year relapse rate with 3 or 4 cycles of HDAC (*n* = 28) compared with one course of HDAC (*n* = 20) in consolidation [30]. A more recent report from CALBG confirmed that among younger patients (<60 years) with CBF-AML, those treated with multicourse HDAC (*n* = 149) in remission were less likely to relapse (*P* < 0.001) compared to ones treated with a single course of HDAC (*n* = 48) [17].

Another way of addressing the optimal dose of post-remission cytarabine is to look at total cumulative dose of cytarabine. The pooled data from multiple German trials could not demonstrate a difference in outcome among cumulative doses of cytarabine that ranged from 20.8 g/m^2^ to 56.8 g/m^2^ [10]. In the CALGB studies, the outcome of patients receiving four cycles of intermediate dose cytarabine (IDAC) consolidation (cumulative infusional cytarabine dose of 8 gm/m^2^) and 3–4 cycles of HDAC (cumulative bolus cytarabine dose of 54–72 gm/m^2^) were similar [17]. A French AML Intergroup study also did not show any difference in outcome between IDAC and HDAC groups [31]. Thus, even though post-remission age-adjusted multi-cycle HDAC is considered standard, the optimal dose of cytarabine remains to be determined.

## Are anthracyclines needed in CBF AML

In the MRC 15 trial, the FLAG-IDA regimen was toxic and resulted in increased rates of myelosuppression and deaths in remission, even though the idarubicin dose was 8 mg/m^2^ (not the usual 12 mg/m^2)^ [25]. In the daunorubicin intensification study conducted by Eastern Cooperative Oncology Group (ECOG) Leukemia committee using a “3 + 7” based regimen, the daunorubicin intensification did not improve the outcome among patients with CBF AML [2].

## Quantitative monitoring of measurable residual disease in CBF AML and MRD-based decision making

The presence of unique transcripts allows for quantitative polymerase chain based (QPCR) monitoring of MRD in CBF AML. The data from multiple studies have confirmed the utility of MRD monitoring by QPCR in identifying differences in patients’ outcomes. [20, 21, 32, 33]. Early transcript reductions at the end of remission induction or after few courses of consolidation are predictive of improved RFS. The MRC group reported that a > 3 log reduction in RUNX1-RUNX1T1 transcripts in the bone marrow in t(8;21)AML at end of induction, predicted for better RFS; the cumulative incidence of relapse (CIR) was 4% among the 47% of patients who achieved this MRD level versus 32% among those who did not. Similarly, *a* < 10 CBFB-MYH11 copy number in peripheral blood (PB), normalized to 10^5^ copies of ABL, in inv(16) predicted for CIR of 21% among the 57% who achieved it versus 50% among the patients who did not. The data from MD Anderson showed that a ≥ 3 log reduction of transcripts in the bone marrow at end of induction and a ≥ 4 log reduction after 2–3 courses of consolidation best predicted for better RFS [20]. While flowcytometry based MRD monitoring could be useful [34], most of the published data supports QPCR based MRD monitoring. One of the limitations in the field is standardization of QPCR MRD monitoring and establishing standards for comparison of QPCR results across institutions and a clear determination of whether the bone marrow or the peripheral blood MRD measurement provided the most useful information.

## Decision making based on MRD

While the prognostic role of MRD monitoring in CBF is well defined, how the data can influence treatment interventions to improve outcome is not clear. Our group reported on the use of hypomethylating agents (HMA) as a potential maintenance strategy in CBF AML, particularly among patients who have persistent MRD after induction/consolidation and patients whose consolidation therapy is curtailed because of older age or adverse events/treatment related serious side-effects [35]. HMA-based therapy can convert a low MRD positive to negative MRD status and/or possibly prevent progression. This strategy may not be effective for high levels of MRD or rapidly increasing MRD.

Investigators from China used allogeneic stem cell transplant (allo-SCT) as an intervention strategy for patients with t(8;21) and suboptimal MRD response by QPCR (defined as <3 log reduction in the bone marrow transcripts). They reported a CIR of 22.1% with allo-SCT versus 78.9% with chemotherapy (*P* < 0.0001; the DFS was 61.7% versus 19.6% (*P* = 0.001) [36]. The MRD-based decision making for suboptimal response to frontline therapy is expected to further evolve in the near future for CBF AML.

## Does additional cytogenetic abnormalities matter

Additional cytogenetic abnormalities involving chromosomes 8, 9, 21, and 22 are common in CBF AML. The presence of trisomy 22 and/or loss of a sex chromosome may predict for better outcome in inv16 AML, but this has not been a uniform finding across studies [5, 17, 31]. The presence of additional cytogenetic abnormalities do not predict for outcome in t(8;21) AML [5].

## The impact of concomitant mutations on outcome in CBF AML

The presence of *KIT* mutations (exon 17) has been associated with a higher relapse rate in CBF AML [37]. However, in our analysis, a suboptimal MRD response by QPCR, and not KIT mutation, was best associated with relapse [20]. In other analyses from larger groups of patients treated with 3 + 7, the presence of multiple kinase mutations (*KIT*, *RAS*, and *FLT3* taken together) [38] and a higher allelic burden (but not presence of *KIT* mutation alone), were associated with higher relapse [39]. The presence of a *KIT* mutation has not been prognostic in an analysis of pediatric CBF AML data [40]. It is of interest that most often, a *KIT* mutation is not detectable at relapse among patients with a baseline *KIT* mutation, raising the question of whether a *KIT* mutation has a driver role in this context.

More contemporary analyses have reported epigenetic mutations (e.g. ASXL2), kinase mutations (JAK2), and cohesion/spliceosome mutations at diagnosis to be associated with a higher relapse rate [14, 16]. Given the strong association of relapse with suboptimal MRD clearance, it will be important to investigate whether any mutation class or combination of mutations is associated with a suboptimal MRD clearance.

## Incorporating kinase inhibition into frontline therapy

Given the prognostic implication of *KIT* mutation, adding potent *KIT* inhibitors like avapritinib or dasatinib into frontline therapy may improve outcome of CBF AML. In a non-randomized trial, the German group reported that the addition of dasatinib into frontline therapy reduced the relapse rate in *KIT*-mutated CBF AML to levels comparable to non-*KIT* mutated CBF [41]. CALGB 10801 trial also reported similar results [42]. A possible rationale to add dasatinib or avapritinib to frontline therapy in all CBF AML derives from the observation that KIT is overexpressed in most CBF AML. Randomized trial data to convincingly support the addition of dasatinib have not been reported.

## The role of allogeneic stem cell transplant in CBF AML

The general consensus is to reserve allo-SCT for relapsed CBF AML. However, an argument can be made for SCT in first remission for patients with suboptimal MRD clearance, where the expected risk of relapse might be high.

## CBF AML in older/unfit patients

Patients older than 60 years comprise about 5-15% of adult CBF AML patients [8, 17, 29] and have a worse OS [8, 17]. A French AML Intergroup study evaluated the outcome of CBF AML among patients 60 years or older (*n* = 147). Cytarabine and anthracycline-based induction chemotherapy resulted in a CR of 80% with the first course, and 88% after a second course. The induction mortality was 10%, and the induction failure/resistance rate was only 2%. Post-remission therapy included either maintenance chemotherapy (low-dose cytarabine, methotrexate, and mercaptopurine; *n* = 72) or intensive consolidation therapy [IDAC/HDAC-based regimen for at least 2 days (*n* = 48), or high-dose melphalan followed by auto-SCT (*n* = 8)]. After a median follow-up of 48 months, the 5-year probabilities of overall survival (OS) and leukemia-free survival (LFS) were 31% and 27%, respectively. The 5-year DFS rate was significantly longer with intensive consolidation compared with low intensity maintenance chemotherapy (*p* = 0.05), and most of the benefit was observed in patients with t(8;21) (*p* = 0.007) but not in patients with inv(16)/t(16;16) (*p* = 0.78) [43]. Therefore, older patients with CBF AML should be offered intensive post-remission chemotherapy if considered fit to receive such therapy. Given high relapse rate, alternate salvage strategies are needed for patients who are not candidates for intensive therapy.

## Treatment of relapsed CBF AML

Salvage intensive chemotherapy has been the norm in relapsed CBF AML. In an analysis of the outcome of 92 patients with relapsed-refractory CBF AML at MD Anderson, the median survival for patients with inv(16) was 15.6 months (range 10.32–20.88 months) and for patients with t(8;21) 9 months (range 3.68–14.32) (*P* = 0.004) [44]. A multivariate analysis showed that t(8;21) was associated with a higher hazard of death after adjusting for age and stem cell transplant (hazard ratio 1.802; *P* = 0.02). In an analysis from the French group, a first remission duration of over 1 year and treatment with regimens incorporating GO were associated with better DFS and OS. The CBF AML cytogenetic subset did not impact outcome [45].

## Secondary or therapy-related CBF AML

While most cases of CBF-AML are de novo, CBF AML can emerge as part of therapy-related AML. While the outcome of secondary or therapy-related acute promyelocytic leukemia (APL), another member of the good-prognosis AML subsets, is similar to de novo APL, the outcome of secondary or therapy-related CBF AML (tCBF-AML) is significantly worse than de novo CBF AML [46, 47]. The presence of a *JAK2* mutation, a poor risk mutation in newly diagnosed CBF AML, is more common in therapy-related CBF AML. In a multi-center data analysis, the t-CBF-AML patients had shorter OS than de novo patients (median 69 vs 190 months, *P* = 0.038) [46].

## Conclusions

**Fig. 1:**
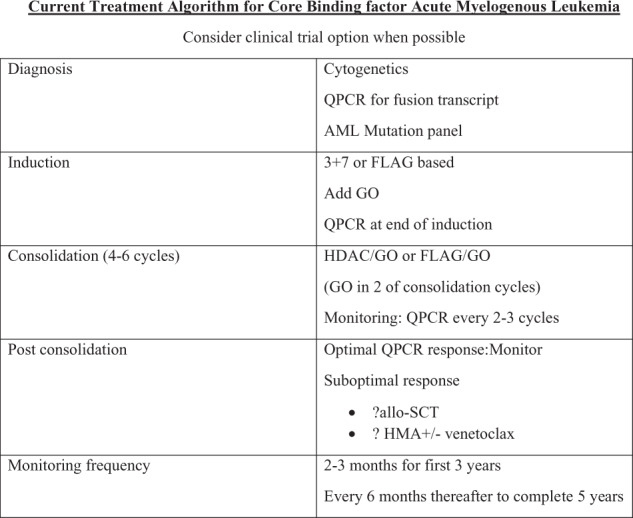
Core binding factor AML treatment and response monitoring algorithm. FLAG fludarabine, cytarabine, GCSF, HDAC high-dose cytarabine, GO gemtuzumab ozogamicin, QPCR quantitative polymerase chain reaction, all-SCT allogeneic stem cell transplant, HMA hypomethylating agent.

Although CBF AML is considered a good-risk AML subgroup, given the results from large co-operative groups and SEER data, improving our current therapeutic strategies with use of existing drugs, can potentially improve the outcome substantially. The incorporation of GO should be widely adopted. Fludarabine and cytarabine based frontline regimens provide effective alternatives to 3 + 7 (Fig. 1). Monitoring of MRD should also be adopted broadly but requires further standardization. Finally, MRD-based treatment interventions/modification should be investigated systematically.
